# Binding affinity prediction for protein–ligand complex using deep attention mechanism based on intermolecular interactions

**DOI:** 10.1186/s12859-021-04466-0

**Published:** 2021-11-08

**Authors:** Sangmin Seo, Jonghwan Choi, Sanghyun Park, Jaegyoon Ahn

**Affiliations:** 1 Department of Computer Science, Yonsei University, Seoul, Republic of Korea; 2 Department of Computer Science and Engineering, Incheon National University, Incheon, Republic of Korea; 3 UBLBio Corporation, 16679 Suwon, Republic of Korea

**Keywords:** Structure-based drug design, Protein–ligand complex, Binding affinity, Attention mechanism

## Abstract

**Background:**

Accurate prediction of protein–ligand binding affinity is important for lowering the overall cost of drug discovery in structure-based drug design. For accurate predictions, many classical scoring functions and machine learning-based methods have been developed. However, these techniques tend to have limitations, mainly resulting from a lack of sufficient energy terms to describe the complex interactions between proteins and ligands. Recent deep-learning techniques can potentially solve this problem. However, the search for more efficient and appropriate deep-learning architectures and methods to represent protein–ligand complex is ongoing.

**Results:**

In this study, we proposed a deep-neural network model to improve the prediction accuracy of protein–ligand complex binding affinity. The proposed model has two important features, descriptor embeddings with information on the local structures of a protein–ligand complex and an attention mechanism to highlight important descriptors for binding affinity prediction. The proposed model performed better than existing binding affinity prediction models on most benchmark datasets.

**Conclusions:**

We confirmed that an attention mechanism can capture the binding sites in a protein–ligand complex to improve prediction performance. Our code is available at https://github.com/Blue1993/BAPA.

**Supplementary Information:**

The online version contains supplementary material available at 10.1186/s12859-021-04466-0.

## Background

Structure-based drug design (SBDD) is widely used for identifying drug candidates. It includes docking-pose evaluation and estimation of the interaction strength between target proteins and small molecules (ligands) [1]. Interaction strength, also known as binding affinity, is calculated using various scoring functions. The stronger the interactions, the more the ligand will affect the physiological function of the target proteins; therefore, ligands that bind strongly to the target protein are selected as drug candidates [2]. Because the predicted binding affinity of the ligand in a library can be used for virtual screening or lead optimization, accurate prediction of binding affinity can reduce the cost of a de novo drug design [3].

Many scoring functions have been proposed to this end, including force-field-based [4, 5], knowledge-based [6, 7], and empirical scoring functions [8, 9]. Empirical scoring functions are known to have the best prediction performance among these three categories [10] and exploit the descriptors of various protein–ligand interactions to calculate a binding affinity score. These descriptors generally include hydrogen bonds with desolvation, van der Waals (vdw), and hydrophobic effects. The estimated coefficient of each descriptor is based on the known binding affinity of protein–ligand complexes. A limitation of the empirical methods, however, is the poor correlation between the experimental and predicted affinity scores. This is primarily because the empirical methods only use few terms related to protein–ligand complexes for easy interpretation of the results, resulting in a failure to describe the actual complexity of protein–ligand complexes [11].

Machine learning-based scoring functions [12–16] have been proposed to overcome the limitations of empirical scoring functions and provide a better prediction performance. These methods exploit various statistical descriptors calculated from information on the chemical and physical structures of known protein–ligand complexes [17]. One representative machine learning-based method is RF-Score [12]. This method represents intermolecular interactions by counting atom pairs with nine heavy-atom types (*C*, *N*, *O*, *F*, *P*, *S*, *Cl*, *Br*, and *I*). RF-Score has shown significant improvement over the existing methods on the PDBbind [18] v2007 benchmark set. Moreover, RF-Score v3 [13], which has six additional features, has achieved a higher prediction accuracy than the original model. Structural interaction fingerprints (SIFt) [14] is another machine learning-based method, which represents the intermolecular interactions in a format that resembles fingerprints. However, a limitation of SIFt is that the number of interaction types is insufficient to handle the complexity of protein–ligand complexes. To overcome this limitation, structural protein–ligand interaction fingerprints (SPLIF) [15] and protein–ligand extended connectivity (PLEC) fingerprints [16] have been proposed. These two methods are based on extended connectivity fingerprints (ECFPs) [19].

Recent advances of deep learning in computer vision have led to the development of deep learning-based scoring functions [20–23]. Compared to traditional machine learning-based methods, deep learning-based methods do not require domain knowledge for feature selection [24] and can identify hidden patterns using nonlinear transformations [25]. Pafnucy [20] and KDEEP [21] are two representative methods that use convolutional neural networks (CNNs). In these two CNN-based methods, each channel is composed of chemical information extracted from a three-dimensional sub-grid for each protein–ligand complex. A problem is that chemical information includes several features such as atomic partial charges, which are calculated using empirical methods such as AM1-BCC [26] and can be incorrect [22].

Fingerprints based on interaction descriptors are an alternative to multidimensional channel representations. However, a limitation of these representations is that they insufficiently consider the complexity of protein–ligand interactions. We defined the descriptors based on the RF-Score features for various interaction patterns. This scenario, however, becomes challenging when considering diversity; an increase in the fingerprint dimensions makes it difficult for the predictive model to capture information that is highly related to binding affinity. In sequence-based binding affinity prediction studies, an attention mechanism was introduced to learn binding sites in the training process from the training data [27, 28]. We introduced an attention mechanism to capture important descriptors for the affinity prediction. Another concern is the lack of distance information in the descriptors used in this study. To supplement the descriptors, we used Vina terms, quantitative numerical values of intermolecular interactions reflecting distance information. This idea is borrowed from RF-Score v3.

**Fig. 1:**
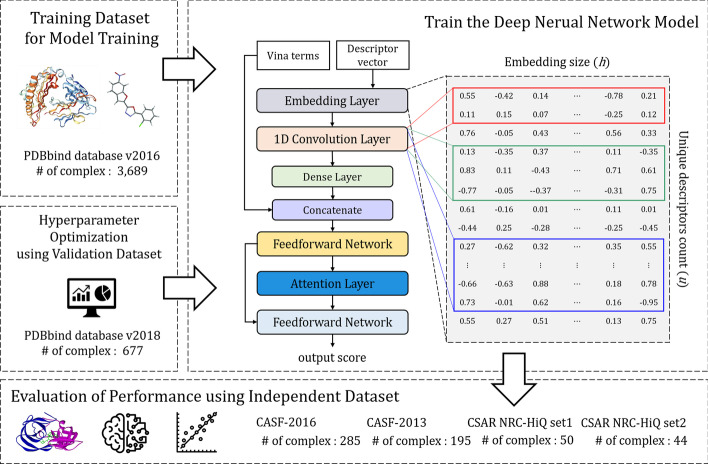
Overview of BAPA experiments. First, we collected training data from the PDBbind database. Second, we constructed a deep learning model that captures local structure patterns that can help predict binding affinity by using convolutional and attention layers. Third, the optimal parameters were found using the validation dataset. Finally, the performance of the model was evaluated using an external test dataset. The numbers (#) of protein–ligand complexes are summarized in each step

This study proposes a deep learning-based model: binding affinity prediction with attention (BAPA), to improve the accuracy of protein–ligand binding affinity prediction. The proposed model has two important features. First, descriptor embeddings that contain embedded information on the local structures of a protein–ligand complex are learnable, which means they are constantly updated to ensure proper embedding of local structures. Second, we introduced an attention mechanism to highlight important descriptors for the binding affinity prediction. A descriptor vector represents information about the local structure of a protein–ligand complex. In BAPA, a convolutional layer transforms the descriptor vectors into latent representations, and an attention layer captures the important descriptors related to binding affinity prediction from these latent representations. This process is illustrated in Fig. 1. When compared with the existing methods on four benchmark datasets, BAPA generally exhibits a better prediction performance.

## Results and discussion

### Model performance evaluation metrics

The performance of the binding affinity models was evaluated using five metrics: mean absolute error (MAE), root mean square error (RMSE), Pearson’s correlation coefficient (PCC), Spearman’s correlation coefficient (SCC), and standard deviation in regression (SD). MAE and RMSE compute the average of errors between the true and predicted affinity scores. The two correlation coefficients measure the linear correlation between true and predicted scores. SD denotes the average distance of the true affinities from the regression line.

### Selection of an optimal number of descriptors

The initial descriptor vector *d* is a sparse vector with the number of descriptor occurrences as its elements. The following experiment was conducted to select only those descriptors that are essential for predicting binding affinity and represent them in a compact vector form. First, we trained a random forest model using training data and sorted the descriptors according to their priorities. Among the 9,333 descriptors, the top 500, 1,000, 1,500, 2,000, 2,500, and 3,000 descriptors were selected. Second, the proposed model was trained using the training dataset, and performance evaluation was conducted according to the number of descriptors using the validation dataset. As shown in Table 1, the best performance was achieved with 2,500 descriptors; therefore, the optimal number of descriptors was set to 2500 (= *u*).

**Table 1:** Performance for different number of descriptors

# Descriptors	# Layer	MAE	RMSE	PCC	SCC	SD
500	3	1.129	1.394	0.667	0.665	1.369
1000	3	1.094	1.366	0.681	0.682	1.346
1500	4	1.094	1.355	0.682	0.684	1.344
2000	4	1.071	1.330	0.696	0.697	1.320
2500	4	1.052	1.314	0.702	0.701	1.309
3000	4	1.061	1.324	0.695	0.694	1.321

### Evaluation of the prediction performance on benchmark datasets

We compared BAPA with four popular prediction models: RF-Score v3 [13], Pafnucy [20], PLEC-linear [16], and OnionNet [22]. All model performances were evaluated with the test datasets after training with the training and validation datasets in this study. The results for the validation dataset are presented in Additional file 1: Table S1.

First, we present the results of the CASF-2016 [29] benchmark set containing 285 complexes. We confirmed that BAPA has the lowest MAE, RMSE, SD, and the highest PCC and SCC, when compared to the other models. For the CASF-2013 [30] benchmark set containing 195 complexes, BAPA outperformed the four baseline models with PCC = 0.771 and SCC = 0.774. Furthermore, when compared to the second-best model, BAPA reduced MAE and RMSE by 0.123 and 0.115, respectively. The results are shown in Table 2. Based on these results, we can say that BAPA has the best performance in terms of the error metrics on the CASF benchmark sets.

**Table 2:** Comparison results using the CASF benchmark datasets

Datasets	Methods	MAE	RMSE	PCC	SCC	SD
CASF-2016	BAPA	1.021	1.308	0.819	0.819	1.247
	OnionNet	1.137	1.542	0.707	0.715	1.539
	Pafnucy	1.327	1.647	0.685	0.681	1.584
	PLEC	1.138	1.454	0.760	0.753	1.412
	RF-score	1.121	1.395	0.812	0.805	1.269
CASF-2013	BAPA	1.170	1.457	0.771	0.774	1.433
	OnionNet	1.423	1.890	0.555	0.605	1.872
	Pafnucy	1.503	1.862	0.592	0.592	1.815
	PLEC	1.246	1.615	0.716	0.724	1.571
	RF-score	1.293	1.572	0.751	0.757	1.487

We performed additional evaluation of the predictive performance using the benchmark set obtained from an external database (CSAR NRC-HiQ [31]). For CSAR NRC-HiQ set1 containing 50 protein–ligand complexes, BAPA had the second-best performance in SCC along with the lowest MAE, RMSE, SD, and the highest PCC. Finally, we presented the results for CSAR NRC-HiQ set2 containing 44 complexes; BAPA showed the best performance in terms of linearity and error metrics. The results are presented in Table 3. These results show that BAPA performs significantly better than the other models in the CSAR NRC-HiQ sets.

**Table 3:** Comparison results using the CSAR NRC-HiQ benchmark datasets

Datasets	Methods	MAE	RMSE	PCC	SCC	SD
HiQ set1	BAPA	1.060	1.453	0.826	0.827	1.329
	OnionNet	1.878	2.462	0.448	0.570	2.105
	Pafnucy	1.832	2.435	0.594	0.561	2.263
	PLEC	1.310	1.772	0.684	0.649	1.717
	RF-score	1.162	1.565	0.799	0.848	1.416
HiQ set2	BAPA	0.982	1.294	0.755	0.782	1.294
	OnionNet	1.313	1.754	0.610	0.685	1.564
	Pafnucy	1.442	1.829	0.722	0.706	1.642
	PLEC	1.057	1.356	0.754	0.747	1.296
	RF-score	1.092	1.430	0.704	0.707	1.402

### Evaluation of model generalization

In machine learning, generalization refers to a models’ ability to adapt to new data that are not used for model training and verifying generalization performance is important for ensuring the practical effectiveness of binding affinity prediction models. Although our proposed model exhibits better predictive accuracy in previous performance tests with various benchmark datasets, it might be difficult to demonstrate its generalization performance with homologous protein–ligand complexes between the training and test datasets.

For rigorous generalization testing, Li et al. [32] proposed a method to construct test datasets using protein-structural and ligand-structural similarity measures. According to the construction process, test datasets include protein–ligand complexes containing either proteins or ligands that have high structural similarity values with those in training datasets, which results in data redundancy, such as for homologous proteins and ligands, which are then excluded from the constructed test datasets. The structural similarity between two proteins is measured using TM-align [33]. TM-align computes a TM-score [34] between 0 and 1; a high TM-score indicates that the two proteins are structurally similar. The structural similarity between two ligands is measured using the Tanimoto coefficient [35]. A ligand is first represented as a binary vector of chemical fingerprints, and the Tanimoto coefficient counts the number of common bits between two ligands and then calculates a similarity value between 0 and 1. A high Tanimoto coefficient indicates the two ligands are structurally similar. The details of the similarity calculations are described in the Methods section.

**Fig. 2:**
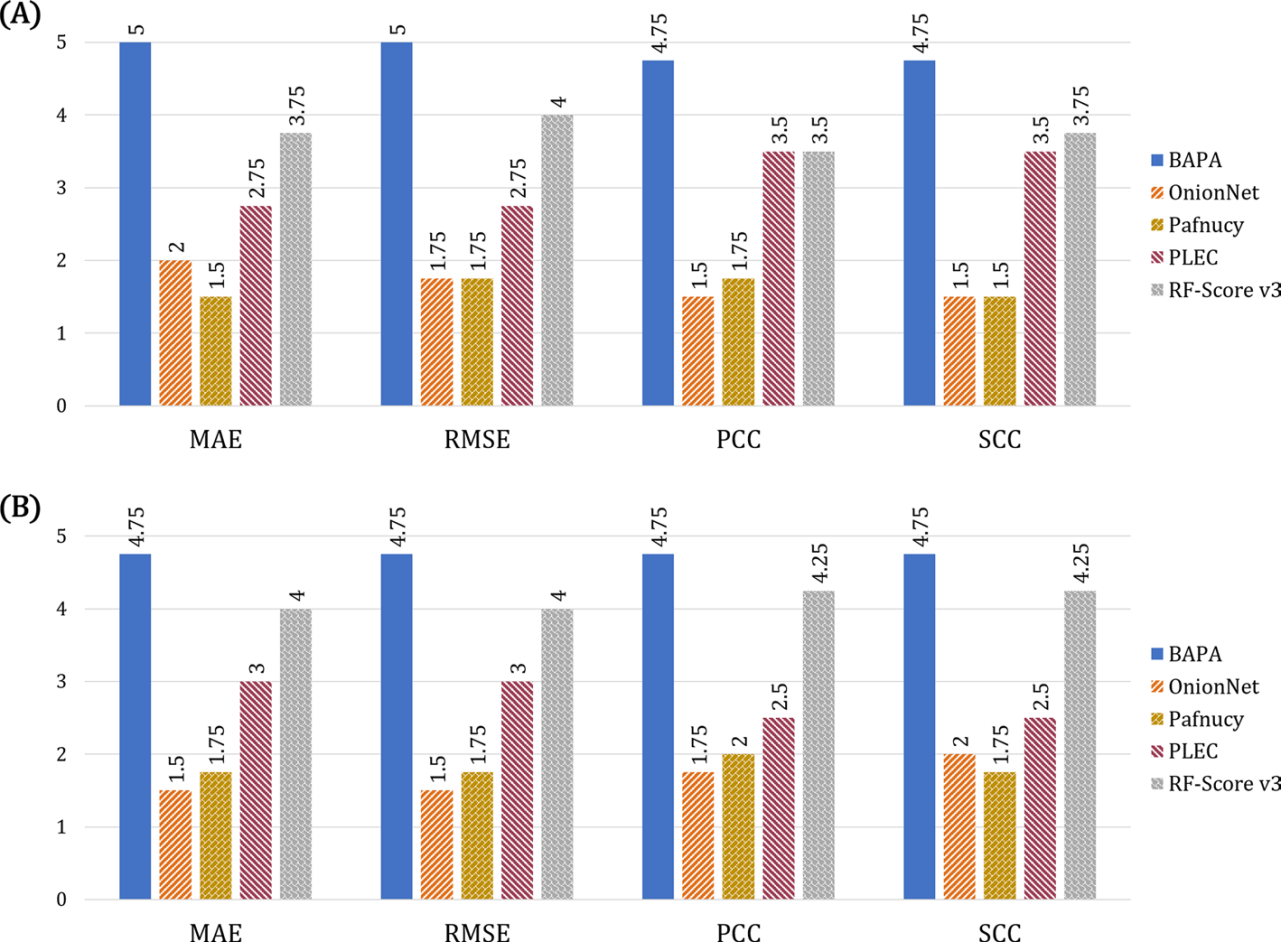
Average ranking comparison results for test datasets. **a** Protein structure generalization test results with lowest pairwise-chains TM-score. **b** Ligand structure generalization test results

BAPA outperformed the existing binding affinity prediction models in generalization tests for proteins and ligands having a low similarity with the training data. To compare the generalization performances, we exploited four test datasets generated from the original benchmark datasets: CASF-2016, CASF-2013, HiQ set1, and HiQ set2, by omitting complexes with high protein-structural or ligand-structural similarities with the PDBbind training dataset. For each metric (MSE, RMSE, PCC, SCC), we evaluated the generalization performances of BAPA, OnionNet, Pafnucy, PLEC, and RF-score on the four test datasets and computed the average rank of each model over the test datasets. Figure 2a, b show the comparison of average model rankings on the protein-structure (lowest pairwise-chains TM-score) and ligand-structure generalization tests, respectively. In both generalization tests, the average ranking score of BAPA was superior to those of the other models. RF-Score and PLEC were observed to be the second and third best models, respectively. The generalization test results of the highest pairwise-chains TM-score are provided in Additional file 2: Fig. S1. The tables are provided in Additional file 1: Table S2–S4.

### Assessment of module importance via ablation test

BAPA showed a superior performance in various benchmark sets by applying 1D convolution to inputs generated from protein–ligand interaction descriptors, adding Vina terms, and using an attention layer. In this architecture, an ablation test was performed using four cases to find the module with the highest influence on model performance. The descriptor vector is denoted as D, the attention layer as A, and Vina terms as V. In the table (D + A) indicates that the experiment was conducted by removing the layer corresponding to Vina terms from BAPA’s architecture (D + V + A). Similarly, (D + V) indicates that the experiment was performed after removing the attention layer.

The worst performance was observed when the descriptors were used alone, as expected. However, contrary to our expectations, the use of Vina terms (D + V) led to a better performance than the use of the attention layer (D + A). In other words, we confirmed that Vina terms have a greater influence on predictive performance improvement than the attention layer. However, the best performance was observed when using both of these modules, confirming that they complement each other. The results are listed in Table 4.

**Table 4:** Ablation test results with CASF-benchmark datasets

Datasets	Methods	MAE	RMSE	PCC	SCC	SD
CASF-2016	D	1.145	1.442	0.771	0.771	1.384
	D + A	1.092	1.389	0.783	0.784	1.351
	D + V	1.075	1.367	0.796	0.790	1.317
	D + V + A	1.021	1.308	0.819	0.819	1.247
CASF-2013	D	1.292	1.579	0.725	0.723	1.550
	D + A	1.270	1.540	0.736	0.731	1.524
	D + V	1.246	1.521	0.755	0.755	1.476
	D + V + A	1.170	1.457	0.771	0.774	1.433

### Analysis of attention vectors

BAPA generally showed a good performance in the test datasets, and we confirmed the attention layer to be an important module for improving the prediction performance in the ablation test. This was presumed to be because BAPA identified the descriptors related to the regions of protein–ligand interactions, for example, binding sites, from the data. To prove this, the attention vectors of two complexes, (PDB ID: 1EBY) and (PDB ID: 3DD0), were calculated, and the attention weights corresponding to the top 10% of each complex were then extracted. This is similar to extracting the descriptors with top 10% attention weights for each complex.

**Fig. 3:**
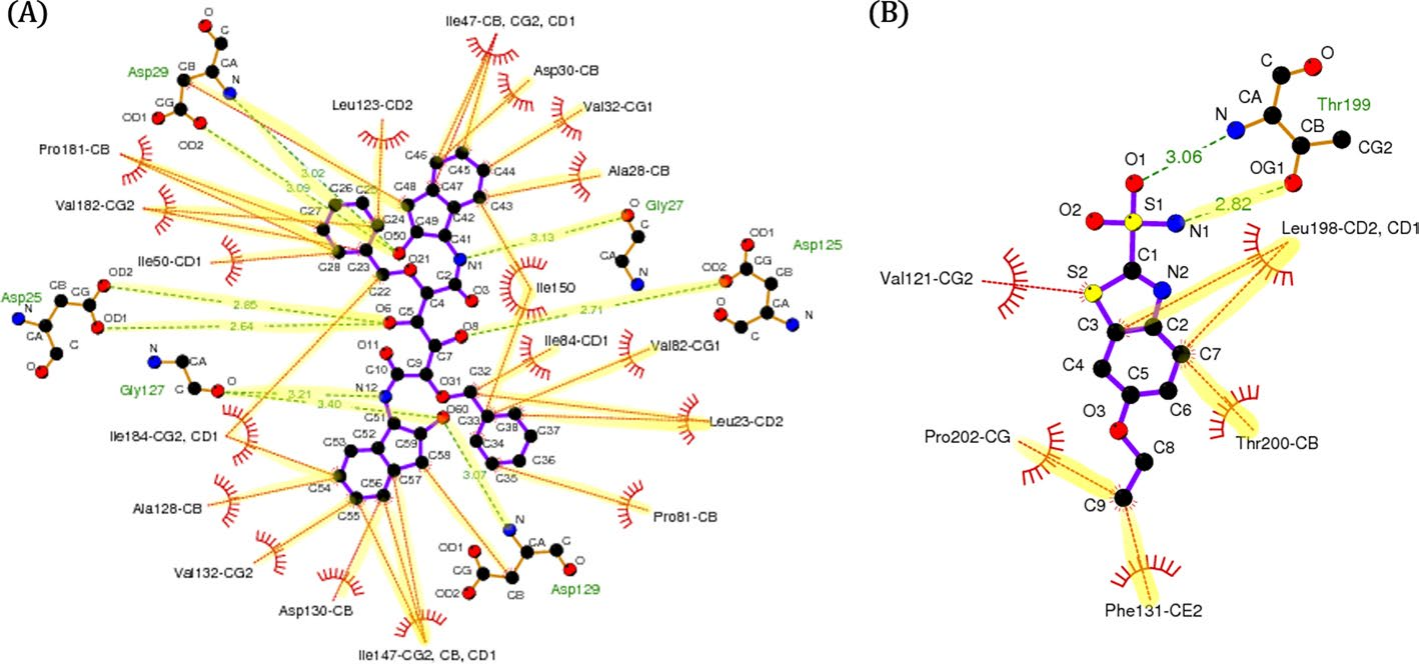
Visualization of interactions sites with high attention score. **a** 1EBY complex, **b** 3DD0 complex. The green dash lines and the brick-red spoked arcs indicate hydrogen bonds and hydrophobic contacts between the two atoms, respectively. Interactions sites captured by BAPA are highlighted in yellow

The 1EBY complex (HIV protease in complex with inhibitor BEA369) has 38 binding site-related interactions based on the sc-PDB database [36]. The inhibitor BEA369 is located at the center and is connected by a purple straight line (ligand bond) in Fig. 3a. The green dashed lines and the brick-red spoked arcs indicate hydrogen bonds and hydrophobic contacts between the two atoms, respectively. For example, (C48, Asp29-CB) connected by a green dashed line indicates a hydrogen bond between the C48 atom of inhibitor BEA369 and the CB atom of aspartic acid, residue 29 of HIV protease. The brick-red spoked arc (C33, Val82-CG1) indicates the hydrophobic contact between the C33 atom of inhibitor BEA369 and the CG1 atom of valine, residue 82 of HIV protease. We confirmed that the extracted top 10% descriptors included all 38 binding sites, which are highlighted in yellow. The results are shown in Fig. 3a. The 3DD0 complex has nine binding sites-related interactions based on the sc-PDB database. We confirmed that the extracted top 10% descriptors include all interactions, except for the following two: (S2, Val121-CG2) and (O1, Thr199-N). The first refers to the hydrophobic contact between the S2 atom of ethoxzolamide and the CG2 atom of valine, residue 121 of carbonic anhydrase 2; the second refers to the hydrogen bond between the O1 atom of ethoxzolamide and the N atom of threonine, residue 199 of carbonic anhydrase 2. These results are shown in Fig. 3b. We can see that BAPA’s attention layer can capture important interaction regions. The figures were plotted using Ligplot + [37].

## Conclusions

In this paper, we proposed BAPA, which can be used for virtual screening and lead optimization in SBDD. The input of the convolutional layers was generated using descriptor information and a learnable embedding matrix. The descriptor is a data structure that contains information about the local structure of protein–ligand complex, and the embedding matrix contains the embedded descriptor information. The embedding matrix is constantly updated for a more appropriate (proper) embedding of the local structure. In addition, an attention mechanism was used to improve the model’s predictive performance. The attention module could identify the important descriptors in a protein–ligand complex and is expected to help researchers design better compounds. BAPA and other existing scoring functions were tested on the CASF-2016, CASF-2013, HiQ set1, and HiQ set2 benchmark sets. BAPA exhibited the best performance on all benchmark sets. In addition, BAPA showed a good generalization performance for a low structural similarity, making it the most suitable method for ligand docking applications that select the ligands “best-fit” to the target protein from a vast chemical space over the four baseline models.

## Methods

### Building dataset and preprocessing

In this study, we used the PDBbind database (version 2016) containing 13,308 protein–ligand complexes. The PDBbind data includes the 3D structure data of protein–ligand complexes and their corresponding experimentally determined binding affinities in terms of dissociation (K_d_), inhibition (K_i_), or half-concentration (IC_50_) constants. Based on the PDBbind database, Wang et al. [10] compiled a *refined set* to provide high-quality complexes and binding data according to the following conditions. First, only complexes with an X-ray crystal structure resolution better than or equal to 2.5 Å were considered. Second, only complexes with known dissociation constants or inhibition constants in the affordable range were considered (pK_i_ and pK_d_ values distributed in the 2–12 range). Third, only non-covalently bound complexes were considered. Fourth, the ligand molecule did not contain any uncommon elements, such as *Be*, *B*, *Si*, and metal atoms. Therefore, only complexes with ligand molecules containing the common heavy atoms (*C*, *N*, *O*, *F*, *P*, *S*, *Cl*, *Br*, and *I*) were considered. Because the quality of a dataset used to develop a scoring function has a significant influence on its performance, we adopted the v2016 *refined set* consisting of 4,507 complexes.

We also adopted the PDBbind v2018 *refined set* (4,463 complexes), CASF-2016 benchmark set (285 complexes), and CASF-2013 benchmark set (195 complexes). The latter two were used only as test datasets for model performance evaluation. To prevent a protein–ligand complex’s simultaneous inclusion in the training, validation, and test datasets, each dataset was constructed according to the following rules. The training dataset comprised 3,689 complexes after removing complexes overlapping with the CASF-2016 and CASF-2013 datasets from the v2016 *refined set*. The validation dataset for model parameter optimization was composed of 677 complexes after removing complexes overlapping with the training, CASF-2016, and CASF-2013 datasets from the v2018 *refined set*. The removal was based on PDB ID.

According to previous studies, training and testing with data from a specific database tend to yield overly optimized results [38–40]. We collected CSAR NRC-HiQ set1 and CSAR NRC-HiQ set2 composed of 176 and 167 complexes, respectively, as external test datasets. For each dataset, we removed complexes overlapping with the training, validation, CASF-2016, and CASF-2013 datasets. Then, the four conditions proposed by Wang et al. were applied. This resulted in 50 complexes for the CSAR NRC-HiQ set1 dataset and 44 complexes for the CSAR NRC-HiQ set2 dataset, which were used as the test datasets. A summary of each dataset is shown in Fig. 1, and the PDB IDs for all complexes in each dataset are provided in Additional file 1: Table S5. All water molecules and cofactors were removed from the crystal structure, and USCF Chimera [41] and Openbabel [42] were used for preprocessing.

### Structure similarity

The protein (ligand) structure similarity of each complex in the test dataset to that in the training dataset is explained here. The structural similarity between the two proteins is calculated by TM-align and expressed as a TM-score. The TM-score ranges between 0 and 1, and higher values indicate that the two proteins are structurally more similar. Since most proteins have multiple chains, all chain structures of each protein were extracted, and their corresponding TM-scores were calculated. The structural similarity between the two proteins was defined as the lowest pairwise-chains TM-score, depending on the chain structure. We also calculated the highest pairwise-chains TM-score. Finally, the protein structure similarity of each complex in the test dataset to the training dataset was defined as the maximum TM-score value. The structural similarity between the two ligands was denoted as a Tanimoto coefficient. The Tanimoto coefficient ranges between 0 and 1, and higher values indicate the two ligands are structurally more similar. As with the proteins, the ligand structure similarity of each complex in the test dataset to the training dataset was defined as the maximum Tanimoto coefficient value. Data on protein structure similarity and ligand structure similarity for each complex are provided in Additional file 1: Table S6.

### Definition of descriptors

**Fig. 4:**
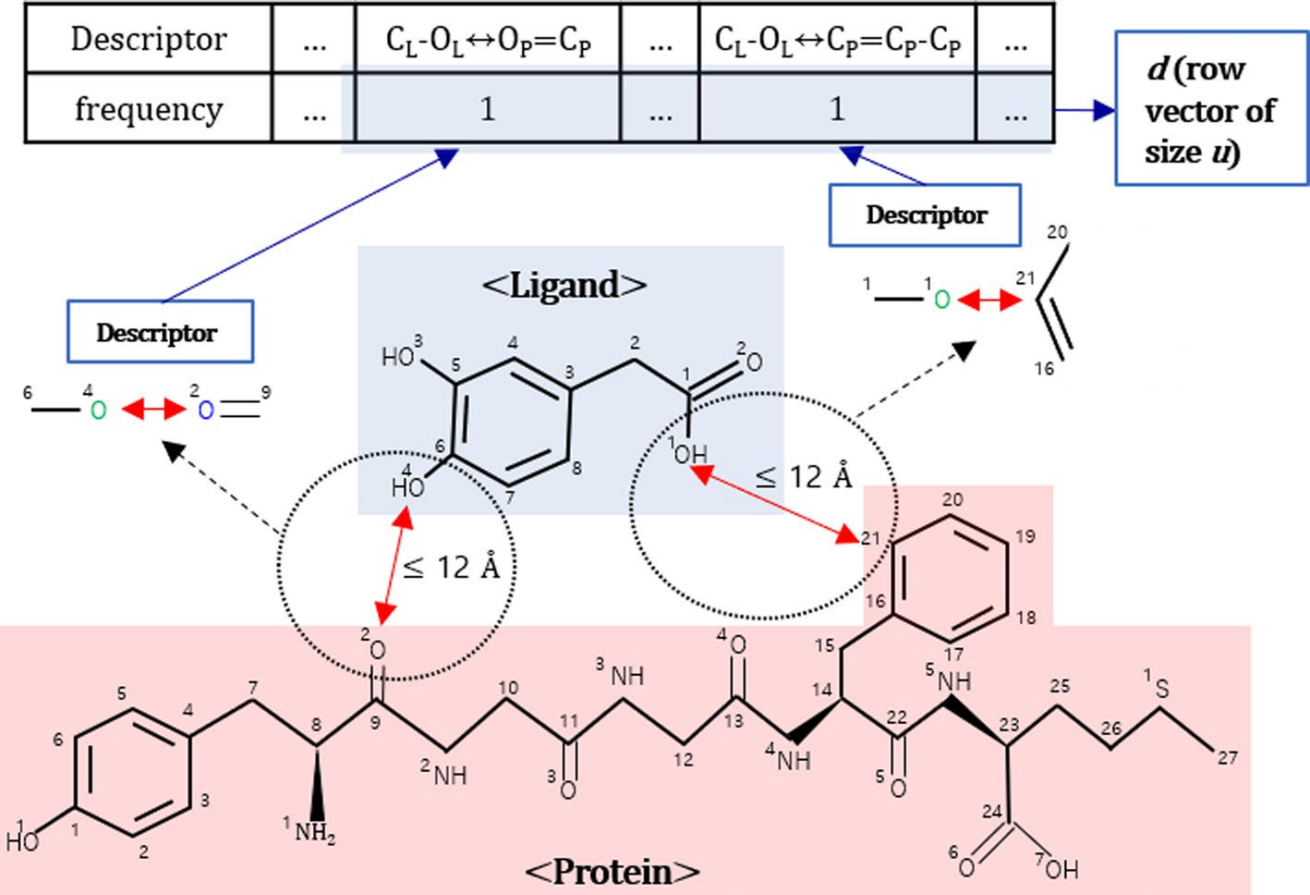
Example of descriptor. There are two atom pairs between which the distance less than 12 $\mathrm{\AA }$, and from which two descriptors can be identified. The frequencies in the protein–ligand complex are counted for all the unique descriptors in the training dataset, with the results being *d*. Note that *OH* is regarded as *O*, and atoms that are not specified are carbon *C*

BAPA’s input, a molecular complex, is represented as a 1D vector, which is calculated based on descriptor information obtained from the training dataset. Using nine heavy atoms commonly observed in protein–ligand complexes, descriptors were generated focused on contacted protein and ligand atom pairs in the molecular complex. Let *L* be a list of heavy atoms in ligands [*C*_*L*_, *N*_*L*_, *O*_*L*_, *F*_*L*_, *P*_*L*_, *S*_*L*_, *Cl*_*L*_, *Br*_*L*_, *I*_*L*_] where *L*[*i*] is the *i*-th atoms type of ligand ($0\le {\varvec{i}}\le 8$). Likewise, let *P* be a list of heavy atoms in proteins [*C*_*P*_, *N*_*P*_, *O*_*P*_, *F*_*P*_, *P*_*P*_, *S*_*P*_, *Cl*_*P*_, *Br*_*P*_, *I*_*P*_] where *P*[*j*] is the *j*-th atom type of protein ($0\le {\varvec{j}}\le 8$). For each *i* and *j*, a set of contacts *X*[*i*][*j*] is defined by:

$${\varvec{X}}\left[{\varvec{i}}\right]\left[{\varvec{j}}\right]=\left\{\left({\varvec{L}}{\left[{\varvec{i}}\right]}_{{\varvec{l}}},\boldsymbol{ }{\varvec{P}}{\left[{\varvec{j}}\right]}_{{\varvec{k}}}\right)\right|{\varvec{d}}\left(({\varvec{L}}{\left[{\varvec{i}}\right]}_{{\varvec{l}}},{\varvec{P}}{\left[{\varvec{j}}\right]}_{{\varvec{k}}})\right)\le {{\varvec{d}}}_{{\varvec{c}}{\varvec{u}}{\varvec{t}}{\varvec{o}}{\varvec{f}}{\varvec{f}}}\}$$

where *L*[*i*]_l_ and *P*[*j*]_k_ are the *l*-th atom of the *i*-th atom type in the ligand and the *k*-th atom of the *j*-th atom type of the protein, respectively. The distance between the protein atom and the ligand atom pair is calculated by Euclidean distance. We used 12 $\mathbf{\AA }$ as *d*_cutoff_, based on previous studies [13, 43]. For example, there are two atom pairs with the distance less than 12 $\mathbf{\AA }$ (in Fig. 4), so ${\varvec{X}}[2][2]=\{({\varvec{L}}{\left[2\right]}_{4},\boldsymbol{ }{\varvec{P}}{\left[2\right]}_{2})\}=\{({{\varvec{O}}}_{{\varvec{L}}4}\leftrightarrow {{\varvec{O}}}_{{\varvec{P}}2})\}$ and ${\varvec{X}}[2][0]=\{({\varvec{L}}{\left[2\right]}_{1},\boldsymbol{ }{\varvec{P}}{\left[0\right]}_{21})\}=\boldsymbol{ }\{({{\varvec{O}}}_{{\varvec{L}}1}\boldsymbol{ }\leftrightarrow \boldsymbol{ }{{\varvec{C}}}_{{\varvec{P}}21})\}.$

The elements of sets *X*[*i*][*j*] form an imaginary edge of which two nodes are ligand and protein atom types. A graph that is extended with one-step neighborhoods from the imaginary edge is defined as a descriptor. The edge between the extended one-step neighborhoods and the imaginary edge has one of five bond types (single, double, triple, amide, and aromatic). Following the previous example, $({{\varvec{O}}}_{{\varvec{L}}4}\leftrightarrow {{\varvec{O}}}_{{\varvec{P}}2})$ is extended to $\mathbf{^{\prime}}{{\varvec{C}}}_{{\varvec{L}}6}-({{\varvec{O}}}_{{\varvec{L}}4}\leftrightarrow {{\varvec{O}}}_{{\varvec{P}}2})={{\varvec{C}}}_{{\varvec{P}}9}\boldsymbol{^{\prime}}$ and $({{\varvec{O}}}_{{\varvec{L}}1}\boldsymbol{ }\leftrightarrow \boldsymbol{ }{{\varvec{C}}}_{{\varvec{P}}21})$ is extended to $\mathbf{^{\prime}}{{\varvec{C}}}_{{\varvec{L}}1}-\left({{\varvec{O}}}_{{\varvec{L}}1}\leftrightarrow {{\varvec{C}}}_{{\varvec{P}}21}\right)-{{\varvec{C}}}_{{\varvec{P}}20}={{\varvec{C}}}_{{\varvec{P}}16}\boldsymbol{^{\prime}}$. Because the order of the bonds (edges) is not considered, ${\boldsymbol{^{\prime}}{\varvec{C}}}_{{\varvec{L}}1}-\left({{\varvec{O}}}_{{\varvec{L}}1}\leftrightarrow {{\varvec{C}}}_{{\varvec{P}}21}\right)-{{\varvec{C}}}_{{\varvec{P}}20}={{\varvec{C}}}_{{\varvec{P}}16}\boldsymbol{^{\prime}}$ and $\mathbf{^{\prime}}{{\varvec{C}}}_{{\varvec{L}}1}-\left({{\varvec{O}}}_{{\varvec{L}}1}\leftrightarrow {{\varvec{C}}}_{{\varvec{P}}21}\right)={{\varvec{C}}}_{{\varvec{P}}16}-{{\varvec{C}}}_{{\varvec{P}}20}\boldsymbol{^{\prime}}$ are the same. Removal of the atom indexes yields two descriptors, $\mathbf{^{\prime}}{{\varvec{C}}}_{{\varvec{L}}}-\left({{\varvec{O}}}_{{\varvec{L}}}\leftrightarrow {{\varvec{O}}}_{{\varvec{P}}}\right)={{\varvec{C}}}_{{\varvec{P}}}\boldsymbol{^{\prime}}$ and $\mathbf{^{\prime}}{{\varvec{C}}}_{{\varvec{L}}}-\left({{\varvec{O}}}_{{\varvec{L}}}\leftrightarrow {{\varvec{C}}}_{{\varvec{P}}}\right)={{\varvec{C}}}_{{\varvec{P}}}-{{\varvec{C}}}_{{\varvec{P}}}\boldsymbol{^{\prime}}$ in Fig. 4. In this way, *u* unique descriptors were calculated from the training dataset, and each protein–ligand complex was represented as a descriptor vector *d* having the frequencies of *u* unique descriptors as elements.

### Autodock Vina-based additional features

BAPA exploits Vina terms that reflect distance information of intermolecular interactions in a protein–ligand complex. We used five additional intermolecular Vina terms and one flexible Vina terms. Intermolecular Vina terms consist of three steric interactions (*gauss*_1_, *gauss*_2_, *repulsion*), *hydrophobic*, and *hydrogen bond* terms. The flexible term is the number of activate rotatable bonds between the heavy atoms of ligand [44].

### Proposed model

#### Overall schema of deep neural network

The proposed model, BAPA, has three kinds of neural network layers (convolutional, attention, and dense) for binding affinity prediction. We designed the model to extract local structure patterns from descriptor vector *d* via the convolutional layer. The latent representation (encoder vector; *e*) of the complex is calculated from the output of the convolutional layer and Vina terms via feedforward network. Based on this information, the attention layer calculates the descriptors important for affinity prediction and yields encoded context vector *c*. Finally, the concatenation of an encoded vector *e* and an encoded context vector *c* is input to the feedforward network to predict the binding affinity. Every layer was activated with the exponential linear unit (ELU) function and the whole network was implemented by TensorFlow (1.12.0). The overall architecture is depicted in Additional file 2: Fig. S2.

#### Convolutional layer with descriptor embeddings

The model starts with the embedding matrix ${\varvec{E}}\in {\mathbb{R}}^{{\varvec{u}}\boldsymbol{ }\times \boldsymbol{ }{\varvec{h}}}$ to transform each descriptor to the corresponding embedding vector ${{\varvec{E}}}_{{\varvec{i}}}\in {\mathbb{R}}^{{\varvec{h}}}$ where *u* is the count of descriptors and *h* is the embedding size. An embedding matrix is initialized by the truncated standard normal distribution. To add local structure information to each descriptor embedding vector, an element of the corresponding descriptor vector (frequency of each descriptor) is multiplied by a weight. Then, the input of the convolutional layer is generated through a dense layer for each column of embedding matrix to which local structure information was added.

To find the pattern in the input, all convolutional layers applied one-dimensional (1D) convolution operation. For example, the first convolutional layer used three filters, and the stride size of each filter is one, so a feature map with a size of (3 × N × 1) is generated. To extract various patterns of the descriptors, five different window sizes (2, 4, 6, 8, and 10) were used, so that five (3 × N × 1) feature maps were generated in the first convolutional layer. Each of these five-feature map passes through the max pooling layer and decreases in size. The depth of the convolutional layer is four and the convolution operation is the same fashion for all convolutional layers, except that the number of filters is six for the second and third convolutional layers, and nine for the fourth convolutional layer. Detailed parameters for the convolutional layers are provide in Additional file 1: Table S7. The results of the fourth convolutional layer are flattened and concatenated, resulting in a single vector of size 513. The single vector and Vina terms are merged into the encoded vector *e*, which is the latent vector of the complex in the feedforward network.

#### Attention layers for important descriptors

In machine translation, the attention mechanism is mainly designed to solve the problem of long-term dependencies when the input sequence is long. When a word is predicted using a decoder, an attention mechanism puts more focus on words that are more related. In this study, we designed the attention layer to focus on more relevant descriptors. The latent representation of the complex (encoded vector; *e*) is input as an attention layer to calculate the contribution of each descriptor to the affinity prediction.

Encoded vector *e* and each row of embedding matrix *E*_i_ are calculated into query vector *q*, key vector *k*_i_, and value vector *v*_i_ through a dense layer. Note that in this study the key vector *k*_i_ and the value vector *v*_i_ have the same value. The similarity between query vector *q* and key vector *k*_i_ ($0\le {\varvec{i}}\le {\varvec{u}}$) is calculated using the inner product. The similarities are transformed into descriptor weights via softmax. The weighted sum of the value vector *v*_i_ over the descriptor weight is used as the context vector. The context vector is input to one dense layer to generate the encoded context vector *c*, which is used to predict the binding affinity together with encoded vector *e*.

#### Feedforward network for binding affinity

The encoded context vector *c*, which is an output of the attention layer, and the encoded vector *e*, are used to predict the binding affinity through the feedforward network consisting of 512, 256, and 128 neurons.

#### Definition of loss function and weight optimization

In the proposed neural network model, the input flows to the output layer in a feedforward fashion. The mean squared error was used as a loss function to train the weights and biases. To prevent overfitting, we applied L2 regularization, so the norm of weights is added to the loss. The Adam optimizer was used for training the network (learning rate 0.005, batch size 256).

## Supplementary Information

**Additional file 1**: **Table S1**. Comparison results using the validation dataset. **Table S2**. Comparison results with lowest pairwise-chains TM-score. **Table S3**. Comparison results with highest pairwise-chains TM-score. **Table S4**. Comparison results with Tanimoto coefficient. **Table S5**. PDB IDs list for all complexes in each dataset. **Table S6**. Structure similarity of the test datasets to the training dataset. **Table S7**. Parameters and architecture in convolutional layers.

**Additional file 2**: **Figure S1**. Average ranking comparison results for highest pairwise-chains TM-Score. **Figure S2** Overview of BAPA.

## Data Availability

The datasets used during the current study are available from http://www.pdbbind.org.cn/.
