# Supplementary material for: Binding affinity prediction for protein–ligand complex using deep attention mechanism based on intermolecular interactions
Source: BMC Bioinformatics. 2021 Nov 8;22:542. doi: 10.1186/s12859-021-04466-0 (PMC8576937; doi:10.1186/s12859-021-04466-0)
Supplement: Supplementary file 2 — Additional file 2: Figure S1. Average ranking comparison results for highest pairwise-chains TM-Score. Figure S2 Overview of BAPA. [file 12859_2021_4466_MOESM2_ESM.docx]

Binding affinity prediction for protein-ligand complex using deep attention mechanism based on intermolecular interactions

Sangmin Seo^1,3^, Jonghwan Choi^1,3^, Sanghyun Park^1*^ and Jaegyoon Ahn^2*^

* Correspondence: [sanghyun@yonsei.ac.kr](mailto:sanghyun@yonsei.ac.kr) and [jgahn@inu.ac.kr](mailto:jgahn@inu.ac.kr)

^1^Department of Computer Science, Yonsei University, Seoul, the Republic of Korea

^2^Department of Computer Science and Engineering, Incheon National University, the Republic of Korea

^3^UBLBio Corporation, Suwon, 16679, Republic of Korea

SI Fig. 1 Average ranking comparison results for highest pairwise-chains TM-score.


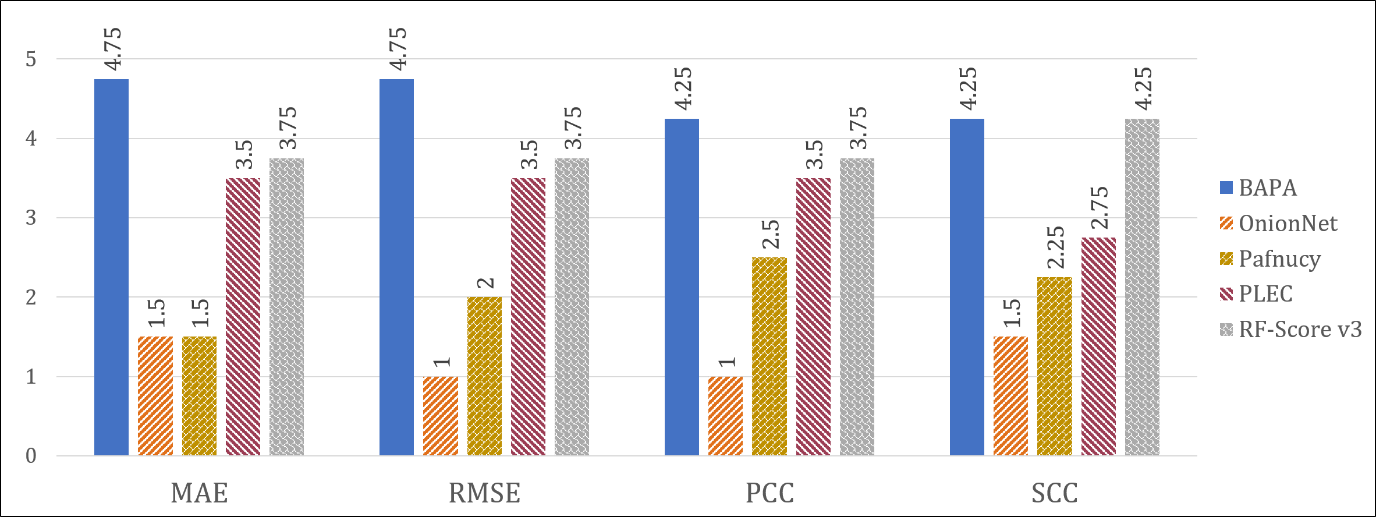

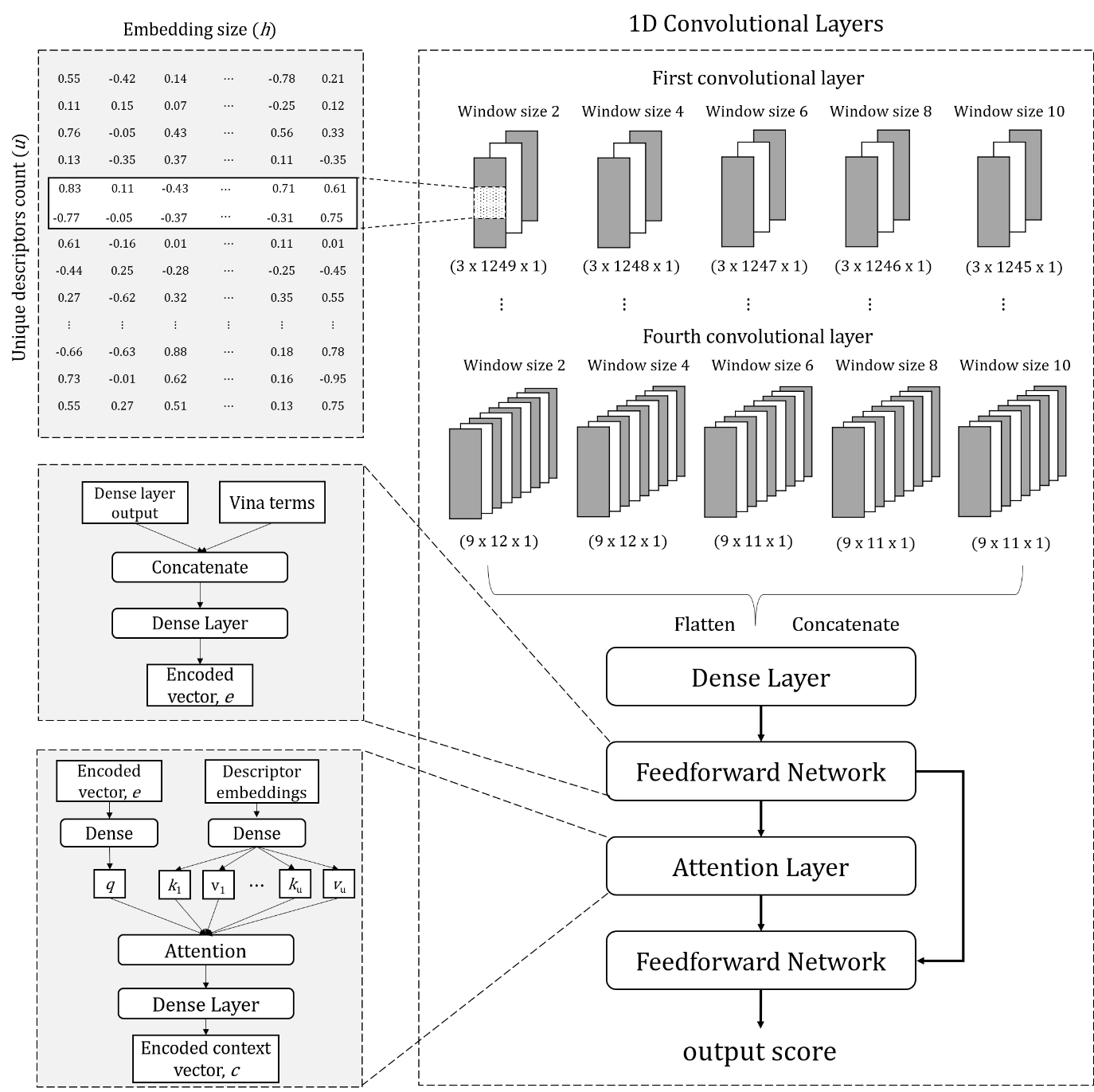


SI Fig. 2 Overview of BAPA. Information about the interaction between the protein and the ligand is represented a single vector of size 513 via convolutional layer and one dense layer. This vector and the Vina terms are used to generate an encoded vector *e*. The priorities (probabilities) of the descriptors are calculated through the attention layer. Finally, two vectors, the encoded context vector *e*, and the encoded vector *c* are input to the feedforward network and used to predict the affinity.
